# Early elevated serum gamma glutamyl transpeptidase after liver transplantation is associated with better survival

**DOI:** 10.12688/f1000research.3316.1

**Published:** 2014-04-03

**Authors:** Edris M Alkozai, Ton Lisman, Robert J Porte, Maarten W Nijsten

**Affiliations:** 1 Surgical Research Laboratory, University Medical Center Groningen, University of Groningen, 9713 GZ Groningen, Netherlands; 2 Hepatobiliary Surgery and Liver Transplantation Section, Department of Surgery, University Medical Center Groningen, University of Groningen, 9713 GZ Groningen, Netherlands; 3 Department of Critical Care, University Medical Center Groningen, University of Groningen, 9713 GZ Groningen, Netherlands

## Abstract

**Background:** Gamma glutamyl transpeptidase (GGT) is a membrane bound enzyme that plays a key role in the synthesis of the antioxidant glutathione. Epidemiological studies have linked high GGT with an increased risk of morbidity and cardiovascular mortality. In contrast, GGT is usually elevated in liver transplant recipients that experience good outcomes.

**Aims**: To study if and how GGT is correlated with mortality following liver transplantation.

**Methods:** We analyzed the prognostic relevance of serum GGT levels during the early and late postoperative period after liver transplantation in 522 consecutive adults. We also studied alanine aminotransferase, aspartate aminotransferase, and total bilirubin levels.

**Results: **Early after transplantation, the peak median (interquartile range) GGT levels were significantly higher in patients who survived more than 90 days compared to non-survivors: 293 (178-464) vs. 172 (84-239) U/l, *p*<0.0001. In contrast, late after transplantation, GGT levels were significantly lower in patients who survived more than 5 years than those who did not ( *p*<0.01). The pattern of GGT levels also differed from those of alanine aminotransferase, aspartate aminotransferase, and total bilirubin early after transplantation, while these patterns were congruent late after transplantation. Kaplan-Meier survival analysis showed that early after transplantation the higher the GGT levels, the better the 90-day survival ( *p*<0.001). In contrast, late after transplantation, higher GGT levels were associated with a lower 5-year survival ( *p*<0.001).

**Conclusions:  **These paradoxical findings may be explained by the time-dependent role of GGT in glutathione metabolism. Immediate postoperative elevation of GGT may indicate a physiological systemic response while chronic elevation reflects a pathological response.

## Introduction

Gamma glutamyl transpeptidase (GGT) is a membrane-bound enzyme that is essential for the synthesis of glutathione (GSH), a key antioxidant ^1^. In clinical practice elevated serum GGT is generally used as an indicator of liver disease, such as biliary obstruction, alcohol consumption, and exposure to certain medical drugs ^1^. Recently, several epidemiological studies have shown that a higher serum GGT level, even within the normal range, is associated with cardiovascular risk factors such as hypertension, hypertriglyceridemia, obesity, type 2 diabetes mellitus and stroke, as well as certain types of cancer ^2– 10^. In contrast to these studies, we observed that after surgery for ruptured abdominal aortic aneurysm ^11^ or after liver resection ^12^, GGT is transiently increased in patients who had a good outcome. In these short-term observational studies GGT level was inversely related to other liver laboratory parameters such as aspartate aminotransferase (ALT), alanine aminotransferase (AST) as well as total bilirubin (TBI) ^11, 12^. We observe that in the early postoperative period after a liver transplantation (LT) a transient gradual increase in GGT also occurs. How early and late postoperative changes in serum GGT are related to survival is not known.

Here we present a study in which we assessed the relationship between early (postoperative day seven) elevated GGT levels with early and late survival (i.e. survival within the 90 days and five years post-LT, respectively). We also evaluated the relationship between late (six months postoperatively) elevated GGT levels with late survival.

In addition, we studied the early and late post-LT kinetics of GGT, AST, ALT, and TBI in patients who survived more than 90 days and in patients who did not. Likewise, these kinetics were compared with long-term survival.

## Materials and methods

### Study population

We conducted a single center cohort study on 522 first liver transplant patients. All LTs performed between January 1990 and August 2009 were included; excluded were pediatric patients (age <17 years), second or subsequent LT, and first LT patients who underwent a re-LT within 90 days of their first LT. Since obstructive mechanisms such as non-anastomotic stricture (NAS), acute graft rejection, and cholestatic disorders might influence GGT and TBI levels, we also specifically repeated our analysis with and without such patients. This study was performed in accordance with Dutch legislation and the local ethical committee guidelines.

### Study variables

Patient characteristics and variables related to the perioperative management and the surgical procedure were obtained from a prospectively collected database. These included age, sex, body mass index, Karnofsky score, indication for LT, preoperative MELD (Model for End-Stage Liver Disease) score (calculated from preoperative laboratory measurements), length of hospital stay, cold ischemia time, warm ischemia time, duration of operation, combined transplant (kidney or lung), acute rejection, graft type, the number of units of allogeneic and autologous red blood cell units (RBC with 1 U containing 300 ml) and fresh frozen plasma (FFP with 1 U containing 250 ml), donor type, type of venous and bile duct anastomosis, and NAS within 90 days and within one year. When necessary, the hospital files were reviewed to complete all relevant clinical parameters.

### The kinetics of serum GGT and other liver function variables early and late post-LT

We studied the levels of serum GGT and other liver variables postoperatively in two ways. Early postoperatively, up to postoperative day (POD) 30, we studied the levels of GGT (reference values; 0–40 U/l), ALT (0–45 U/l), AST (0–40 U/l), and TBI (0–17 µmol/l) over time in patients who survived more than 90 days after LT compared to those who did not. Late postoperatively (i.e. 90 days and beyond), we evaluated the levels of these variables at three months, six months, and one year in patients who survived more than five years compared to those patients who did not.

### Survival analysis and elevated GGT early and late following LT

To evaluate the clinical relevance of early and late elevated GGT levels, we generated tertiles of low, intermediate, and high GGT levels based on equal percentiles. GGT levels at POD 7 were used to study the relationship between early elevated GGT levels and both 90-day and five-year survival. GGT levels at six months following LT were used to study the relationship of late elevated GGT with five-year survival. The last observation date for the status of patient survival for the study cohort was August 23, 2012.

### Statistical analysis

Statistical analyses were performed using the statistical software package SPSS 20 (IBM SPSS, Chicago, IL). Categorical variables are shown as numbers and percentages. Continuous variables are presented as means with standard deviation (SD) or as medians with interquartile range (IQR) based on their distribution. Continuous variables that were not normally distributed were compared using the Mann Whitney U test. We studied early LT mortality based on GGT levels at POD 7 as a categorical variable, using tertiles (low, intermediate, and high). Similarly, we assessed the late LT mortality based on GGT levels at six months post-LT using tertiles.

Patient survival was analyzed with Kaplan-Meier analysis and the differences between the groups were assessed with the log-rank test. A *p*<0.05 was considered statistically significant.

## Results

### Study population

We performed a total of 968 consecutive LTs in our center between January 1990 and August 2009. After excluding pediatric LTs (age <17 yr; n=290), patients who were re-transplanted within the 90 days of their first LT (n=39), second or subsequent LTs (n=101), patients with a lack of follow up data (n=11), and patients that died intraoperatively (n=5) due to brain death, cardiac failure, or uncontrollable bleeding, 522 patients were included in our analyses. The median age was 48 years (37–56), 54% of the patients were males, mean (SD) BMI was 24.8 (± 5.3), median Karnofsky score was 60 (30–70) and median MELD score was 17 (13–24) for the study population. Indications for LT were post necrotic cirrhosis (49%), cholestatic liver disease (30%), metabolic disease (10%), acute liver failure (7%), and miscellaneous (5%). Patient characteristics and the surgical variables of the entire group of 522 patients are summarized in Table 1.

**Table 1.** Characteristics of study population.

Demographic characteristics	Total n=522
**Recipient variables**	
Gender, male	283 (54%)
Age, years, median (IQR)	48 (37–56)
BMI, mean (SD)*	25 (5)
Karnofsky score, median (IQR)	60 (30–70)
MELD, median (IQR)*	17 (13–24)
**Indication for LT**	
Post necrotic cirrhosis	254 (49%)
Cholestatic liver diseases	155 (30%)
Acute liver failure	38 (7%)
Metabolic disease	51 (10%)
Miscellaneous	24 (5%)
**In hospital stay, days (IQR)**	30 (21–46)
**ICU stay, days (IQR)**	3 (2–7)
**Transplantation variables**	
Operation length (minutes), mean (SD)	581 (119)
WIT (minutes), mean (SD)	53 (14)
CIT (minutes), mean (SD)	573 (192)
**Combined transplant (kidney or lung)**	18 (3%)
**Acute 90 day rejection**	178 (34%)
Mild, not treated	75 (42%)
Moderate severe, treated	103 (58%)
Graft type*	
Full size	503 (97%)
Split or reduced size	18 (3%)
ABO compatibility*	
Identical	489 (95%)
Compatible	28 (5%)
**Blood loss (liters), median (IQR)**	5.0 (2.1–8.5)
**RBC transfusion (unit=300 ml), median (IQR)**	6 (2–11)
Donor type*	
Heart beating	435 (91%)
Non-heart beating	38 (8%)
Living donor	3 (1%)
**Venous anastomosis**	
Piggyback	302 (58%)
Classic	220 (42%)
**Bile duct anastomosis**	
Duct to duct	456 (87%)
Rou-x-en Y	61 (12%)
Duct-duodenostomy	5 (1%)
**Non-anastomotic stricture,**	
90-day, yes	30 (6%)

*Some variables were not available for all patients. CIT, cold ischemia time; ICU, intensive care unit; IQR, interquartile range; INR, international normalized ratio; SD, standard deviation; WIT, warm ischemia time

###  Mortality rates and major causes of early mortality

The overall mortality within 90 days, one year, and five years for the study cohort was 8%, 12%, and 21%, respectively. Sepsis was the major cause of mortality (37%) within 90 days followed by multi organ failure (14%), and brain death (9%). Table 2 details all causes of mortality within the first 90 days.

**Table 2.** Causes of mortality within the 90 days after LT.

Cause of death	Frequency n=43 (%)
Sepsis	16 (37%)
Multi organ failure	6 (14%)
Brain death (metabolic and hepatic encephalopathy)	4 (9%)
Transplant failure	3 (7%)
Pulmonary embolism	3 (7%)
Bleeding	3 (7%)
Cardiac failure	3 (7%)
Respiratory failure	1 (2%)
Graft vs. host disease	1 (2%)
Unspecified	3 (7%)

### Early post-LT GGT and other liver variables and early mortality

**Figure 1.**
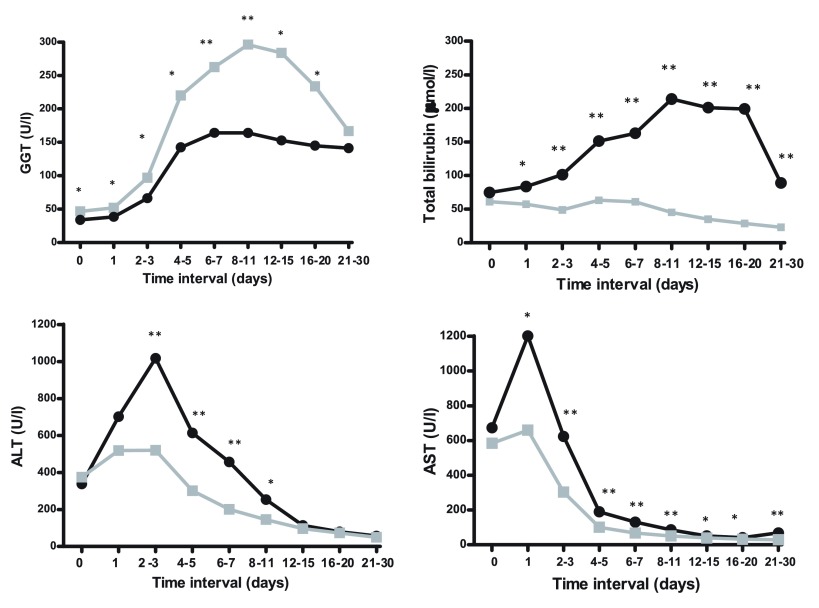
Evaluation of early post-LT laboratory variables (i.e. 0–30 POD) in early mortality. Curves represent patients who survived more than 90 days (gray) and those who did not (black). Median values are shown. * *p* < 0.05; ** *p* ≤ 0.001. GGT, gamma glutamyl transpeptidase; ALT, aspartate aminotransferase; AST, alanine aminotransferase; TBI, total bilirubin.

Early postoperative laboratory variables are shown in Figure 1. Postoperatively, GGT levels increased gradually, reaching a maximum at POD 9 and decreased thereafter. Notably, the increase in GGT levels was significantly more pronounced, i.e. deviated more from the normal range, in patients who survived more than 90 days, as compared to those who did not: 297 (178–464) vs. 172 (69–271) U/l, *p*<0.0001, respectively. This pattern was different from that of postoperative levels of TBI, AST, and ALT. TBI was consistently and significantly lower in patients who survived more than 90 days following LT as compared to those who did. AST and ALT levels increased rapidly until POD 1 and POD 2, respectively, followed by rapid normalization thereafter. Contrary to GGT, the peak levels of AST and ALT were significantly lower in patients who survived more than 90 days following LT as compared to those who did not: AST 659 (326–1267) vs. 1201 (451–1990) U/l, *p*=0.01 *,* and ALT 527 (280–1080) vs. 1082 (529–2631) U/l, *p*=0.001, respectively.

Thirty patients developed NAS within the 90 days post-LT. Since these patients may present with abnormal high TBI and GGT levels, we repeated the analysis with exclusion of these 30 patients with no significant affect on the GGT levels (graphical representation not shown). Also exclusion of patients who were treated for developing an acute rejection (n=103) and those who underwent LT for cholestatic liver disease (n=155) did not significantly affect the outcomes (graphical representation not shown).

### Late post-LT GGT and other liver variables and mortality

**Figure 2.**
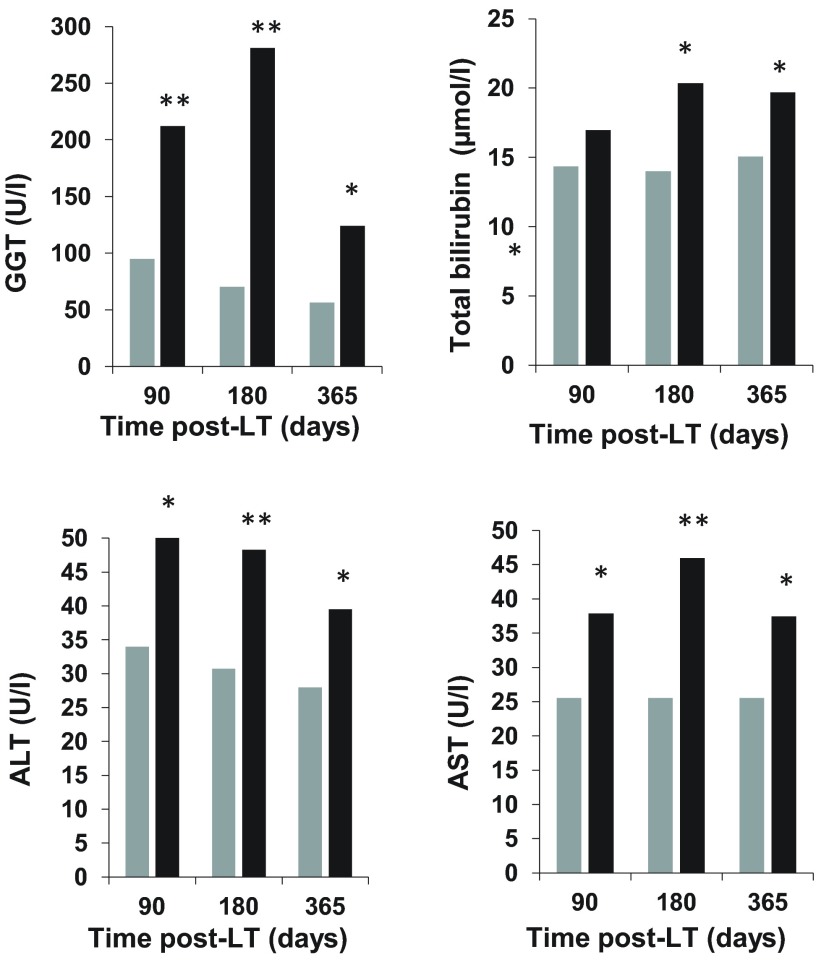
Evaluation of three, six, and twelve months post-LT laboratory variables in late mortality. Bars represent patients who survived more than five years (gray) and those who did not (black). Median values are shown. * *p* < 0.05; ** *p* ≤ 0.001. GGT, gamma glutamyl transpeptidase; ALT, aspartate aminotransferase; AST, alanine aminotransferase; TBI, total bilirubin.

Late postoperative laboratory variables are shown in Figure 2. We studied the changes in GGT levels over time in patients who survived more than five years as compared to those who did not survive. Compared to patients who died within five years post-LT, those who survived more than five years had significantly lower GGT at three months post-LT; 95 (42–244) vs. 212 (92–400) U/l, *p*=0.001, six months post-LT; 70 (31–189) vs. 281 (103–438) U/l, *p*<0.001 *,* and 1 year post-LT; 57 (25–153) vs. 124 (45–431) U/l, *p*=0.003, respectively. Notably, late post-LT the GGT levels showed the same patterns as TBI, AST, and ALT, i.e. higher levels in non-survivors.

### Kaplan-Meier survival analysis

**Figure 3.**
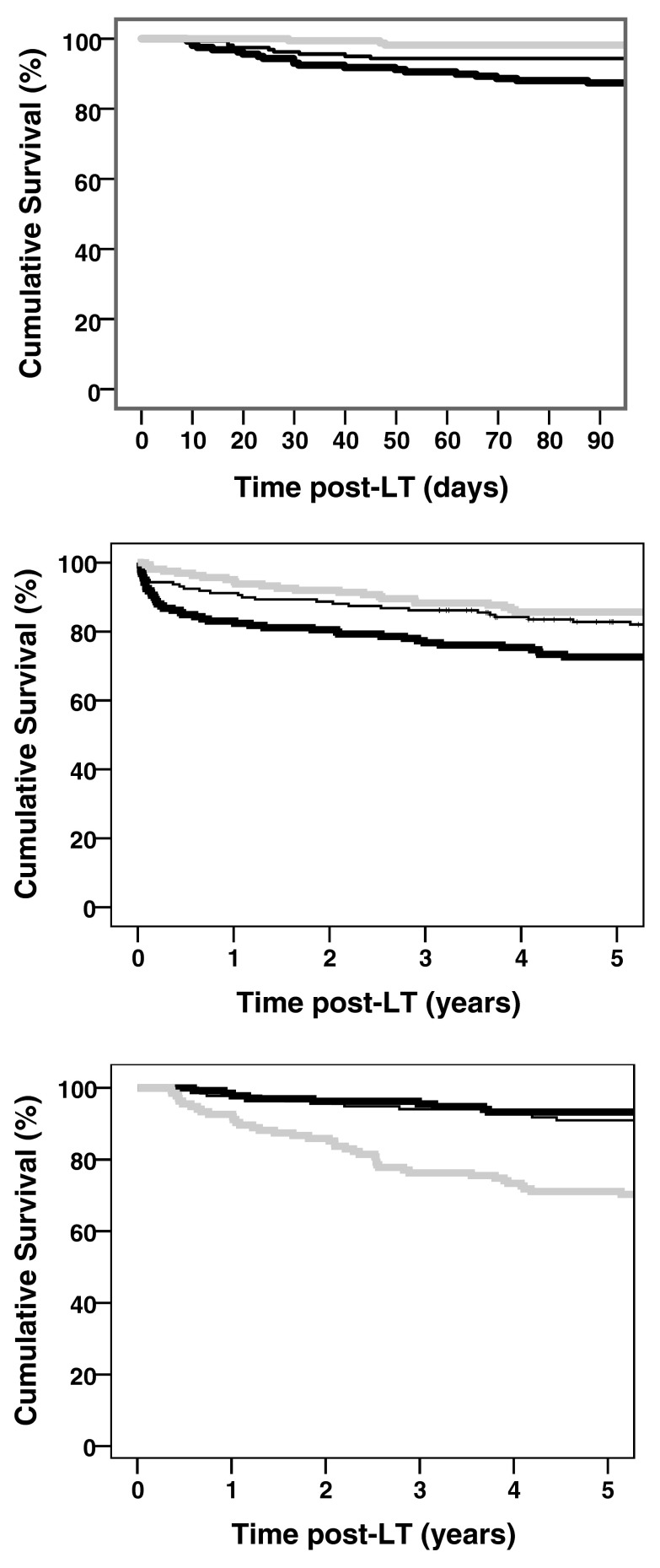
Kaplan–Meier analysis showing the opposite relationships of early and late GGT levels with early and late mortality. Panels A and B represent survival analysis post-LT in relation to GGT tertiles at POD 7, within 90 days and five years respectively (p=0.003). Panel C demonstrates five-year survival in relation to GGT tertiles at six months post-LT (p<0.001). Curves represent low (thick black), intermediate (thin black), and high GGT (gray).

We also studied the clinical relevance of early versus late elevated GGT levels with Kaplan-Meier survival analysis. Figure 3 plots the overall 90-day and 5-year overall survival for the study cohort based on GGT-tertiles. A high GGT level at POD 7 was significantly associated with better early survival following LT ( Figure 3A). The overall 90-day survival was 98% for high GGT (≥ 351 U/l), compared to 94% for intermediate GGT levels (188 and 350 U/l), and 87% for the low GGT (≤ 187 U/l), at POD 7 ( Figure 3A). Similarly, five-year overall survival was 86%, 83%, and 73% for high, intermediate, and low GGT at POD 7 ( *p=*0.003; Figure 3B), respectively. Remarkably, the differences in five-year survival mainly developed during the first three months post-LT with almost no difference in survival curves thereafter ( Figure 3B). In sharp contrast with early GGT, a high GGT level six months post-LT was associated with lower five-year survival ( Figure 3C). The overall survival within five years was 71% for elevated GGT (> 163 U/l), compared to 91% for intermediate GGT levels (44 and 163 U/l), and 93% for the low GGT (< 43 U/l), *p*<0.001.

Serum gamma glutamyl transpeptidase levels post-liver transplantation and survival data “appr_F1000_GGTpaper_perioperativeCharacteristics.sav” The file contains data concerning the table 1 in the associated article including patients and surgical characteristics for the entire study population. Excluded was the indication for liver transplantation (LT).
“appr_F1000_GGT_paper_LT_Indication.sav” The file contains data concerning the indications for LT described in table 1 in the associated article.
“appr_F1000_GGTpaper_mort90d_reason.sav” The file contains data concerning table 2 in the associated article. Contained are causes of early mortality (i.e. within the 90 days post-LT).
“appr_F1000_GGTpaper_EarlyLabMortality.sav” The file contains data concerning figure 1 in the associated article. Contained are the laboratory values for gamma glutamyl transpeptidase (GGT), total bilirubin, aspartate aminotransferase (ALT), and alanine aminotransferase (AST), and the status of survival within the 90 days post-LT.
“appr_F1000_GGTpaper_LateLabMortality.sav” The file contains data concerning figure 2 in the associated article. Contained are the laboratory values GGT, total bilirubin, ALT, and AST, and the status of survival within the 5 years post-LT.
“appr_F1000_GGTpaper_KaplanMeijerF3.sav” The file contains data concerning figures 3A, B, and C in the associated article. Contained are observation period, survival status, and the GGT levels at postoperative day (POD) 7 and POD 180 as a categorical variable.

## Discussion

In this study, we evaluated the changes in GGT over time following liver transplantation and the clinical relevance of these changes for early and late survival. We found that a transiently elevated GGT early after LT was associated with increased survival rates within the first 90 days. In contrast, late elevation of GGT was associated with decreased five-year survival following LT. Although the early GGT elevations was also associated with five-year survival, this difference mainly developed during the first 90 days post-LT.

This peculiar effect was not observed for TBI, AST, and ALT since higher levels for these parameters at POD 7 and six months were associated with increased mortality at both 90 days and five years after LT.

To our knowledge, this is the first study showing the short and long term kinetics of GGT and the clinical relevance of an early elevated serum GGT in LT recipients. Previously, we have reported improved outcome in patients with significantly increased levels of GGT in the early post-operative period following liver resection ^12^ and surgical repair of a ruptured abdominal aortic aneurysm ^11^. However, those studies were not designed to address changes in GGT progression over time.

While we acknowledge that association does not necessarily indicate causation, these data support the hypothesis that high GGT in an early post-LT setting may be a marker of some protective process.

Although the precise mechanism responsible for an elevated serum GGT early after LT is yet to be determined, experimental studies have demonstrated that cellular GGT modulates crucial redox-sensitive functions, such as antioxidant and antitoxic defenses, cellular proliferation, and apoptotic balance ^13^. Cellular GGT is a key enzyme in the gamma-glutamyl cycle resulting in production of intracellular GSH ^14– 16^, an important antioxidant agent that protects the cells against reactive oxygen species (ROS) ^17– 19^. GSH has been shown to protect the liver against ischemia reperfusion injury in animal models ^16, 20, 21^. Hepatic ischemia can cause elevation of serum GGT with peak blood levels within 20 and 30 hours after restoration of hepatic arterial blood flow ^18, 24^. Reperfusion is associated with a surge of ROS, which may overwhelm host natural antioxidant defenses ^21^. The oxidative stress from the ROS formed after reperfusion may lead to increased cellular death by damaging membrane lipids through peroxidation, disrupting normal enzymatic activities, and diminishing mitochondrial oxidative metabolism ^22^. Cardin and colleagues ^23^ studied oxidative stress in patients with chronic hepatitis C virus infection. Surprisingly, the authors observed an association between GGT and 8-hydroxydeoxyguanosine (8-OHdG), a marker of oxidative DNA damage. Patients who had a high level of 8-OHdG had significantly higher GGT levels but normal ALT levels ^23^.

Thus, a transient increase in GGT level post-LT may reflect the host compensatory mechanism against oxidative stress and toxic metabolites generated by hypoxia, reperfusion, and surgical stress ^21^. Therefore, an increased GGT early after LT may reflect the ability of the host to initiate an appropriate systemic response.

Another explanation for the elevated serum GGT in the early post-LT period that has been suggested relates to liver regeneration. Eisenbach and colleagues ^25^, showed that an early increase in serum GGT after LT was associated with a better outcome and the authors reasoned that this rise could be due to liver regeneration. Although the liver might regenerate to some extent after LT, there is no conclusive evidence to support this hypothesis. As we mentioned earlier, we observed a transient increase in serum GGT levels in patients who survived a surgical repair for a ruptured abdominal aortic aneurysm ^11^. In the latter group, it is less likely that significant liver regeneration occurs.

Contrary to early elevated GGT, but in line with published literature ^2– 10^, we observed that a late (i.e. six months post-LT) elevated GGT was significantly associated with decreased survival within the 5-years following LT. Although the finding that normal GGT levels in the late post-operative period is predictive of good outcomes is obvious and intuitive, the contrasting influences of GGT levels between early and late post-LT periods on survival may be compatible with the physiologic function of intracellular GGT. Notably, at three months, six months, and one year post-LT, the relative increase in serum GGT was two to four times higher in patients who did not survive for more than five years compared to those who did survive. This proportion was much higher than that of AST, ALT and TBI, which might imply that an elevated serum GGT is not only a marker of harm to the liver but it could be seen as a systemic response to harmful environmental factors. Indeed, in two studies, Lee and colleagues ^26, 27^ postulated that serum GGT in the general population might be a marker of increased exposure to environmental stress, internal xenobiotics, or other unknown factors that cause oxidative stress in the long run.

To avoid possible bias we performed our analysis after excluding obstructive mechanisms such as NAS, cholestatic disorders, and acute graft rejection early postoperatively. Exclusion of these cases did not change our results significantly, suggesting that the elevation in serum GGT early post-LT is independent of obstructive disorders.

A practical clinical consequence of our findings may be that care providers in hospitals should realize that an abnormally high GGT early post-LT is not a cause for alarm or specific diagnostic procedures. In fact, a GGT activity four to five times above the normal range during the second post-operative week might even be considered beneficial.

We acknowledge some considerable limitations in this study. First, due to the retrospective design of the study we can only identify association rather than causation. This could only be established by specific (intervention) studies that measure the interaction between serum GGT and ROS markers post-LT. However, there is a strong correlation between GGT and oxidative DNA damage in cirrhotic patients ^23^. Next, we cannot entirely exclude that liver regeneration plays a role in the early post-LT rise of GGT levels since GSH and to some extent GGT are mainly produced by the liver ^1^. Hence a high serum GGT in patients who survived more than 90 days can also be a reflection of a well-functioning graft in LT patients. It will be important to understand the relationship of serum GGT and cellular GGT in the period immediately after surgery. Besides, cumulative evidence suggests that there is a relationship between the induction and release of ROS and ischemia reperfusion injury after other types of abdominal surgery ^18, 21, 24^.

In conclusion, an elevated GGT level early after LT was associated with a better short-term outcome. However, chronically elevated GGT was associated with poor long-term outcome in the outpatient setting after LT. This peculiar switch in the prognostic meaning of GGT may result from the superposition of several mechanisms. Apparently, higher expression of GGT protects in the acute phase but reflects chronic damage over the long term.

## Data availability

figshare: Serum gamma glutamyl transpeptidase levels post-liver transplantation and survival data, http://dx.doi.org/10.6084/m9.figshare.900343 ^28^
