# Supplementary material for: Early elevated serum gamma glutamyl transpeptidase after liver transplantation is associated with better survival
Source: F1000Res. 2014 Apr 3;3:85. [Version 1] doi: 10.12688/f1000research.3316.1 (PMC4097359; doi:10.12688/f1000research.3316.1)
Supplement: Peer review history

# Peer review: Referee response for version 1

**DOI:** 10.5256/f1000research.3557.r5247

**Published:** 2014-07-10

**Authors:** Peter Jansen

**Affiliations:** 1 Division of Gastroenterology and Liver Diseases, University of Amsterdam, Amsterdam, Netherlands

This is an interesting paper providing novel insights into the value of traditional serum markers. It is quite true that the understanding of elevated GGT values is very limited, despite the fact that this value is one of the most requested 'liver function tests'. Most clinicians who request this test probably have no clear understanding what is being tested. It is very interesting that elevated GGT shortly after liver transplantation is associated with a better outcome. 

The title, abstract, data section, statistics and figures of the paper are OK, the discussion section could be a little more to the point.

In my opinion there might be two reasons for an elevated GGT early after transplantation: 1) Elevated GGT is associated with oxidative stress, and some oxidative stress may prompt the hepatocyte to activate innate protective mechanisms; 2) Elevated GGT after transplantation may be an indicator of good early secretory function of the liver. GGT in part comes from the surface of the bile ducts and is released from the anchor which attaches this enzyme to the cell surface, by bile acids in bile. These same authors have shown that immediately after transplantation the bile acid/phospholipid ratio in bile is elevated and (as in MDR3 deficient/LPAC patients) this leads to elevated GGT. Thus elevated GGT immediately after transplantation indicates an active bile flow. This protects hepatocytes from cytotoxic bile acids. Later after transplantation when the BA/PL ratio has normalized, bile has lost some of its detergent action, with lower basal serum GGT levels as a result. When GGT is elevated at this stage it has become the traditional indicator of liver damage associated with a bad outcome, as the authors indicate.

If the authors agree with this reasoning they could include some of these thoughts in the discussion section, in order to provide a little bit more insight into why elevation under some conditions may be beneficial as this, in my opinion, is the main and novel message of this paper.

I have read this submission. I believe that I have an appropriate level of expertise to confirm that it is of an acceptable scientific standard.

# Peer review: Referee response for version 1

**DOI:** 10.5256/f1000research.3557.r4362

**Published:** 2014-04-22

**Authors:** Michael Olausson

**Affiliations:** 1 Department of Surgery, University of Gothenburg, Gothenburg, Sweden

This is a very useful paper on Gamma Glutamyl Transpeptidase (GGT or just GT) - a test used in many liver transplant units and in hepatologist outpatient clinics. Its main use is the proposed correlation to liver damage - may it be cholestasis (often in combination with ALP elevation), rejection or infection. It is also used in alcoholic abuse monitoring. The early kinetics after liver transplantation are less known, and this paper gives valuable information regarding this.

 The authors found that livers with longterm function had an early raise in GT levels, not indicating signs of liver damage, while late raised levels showed the more expected liver damage as reason. Possible causes for early elevated levels are discussed, and a very relevant review article written by Whitfield (2001) is recommended.

**Title:**

The title reflects the take home message.

**Abstract:**

I think the abstract should stress the connection to oxidative stress mentioned in the review article more, since this seems to be a very plausible mechanism.

**Methods:**

The study is well conducted and patient selection well explained. The validity check of the findings by excluding NAS and cholestatic patients strengthen the results.

**Conclusions:**

The conclusions could merit from referring to the correlation to oxidative stress more. It is not *proven* that higher early GT actually protects the liver - it could be a surrogate marker for a liver in less oxidative stress.

I have read this submission. I believe that I have an appropriate level of expertise to confirm that it is of an acceptable scientific standard.
